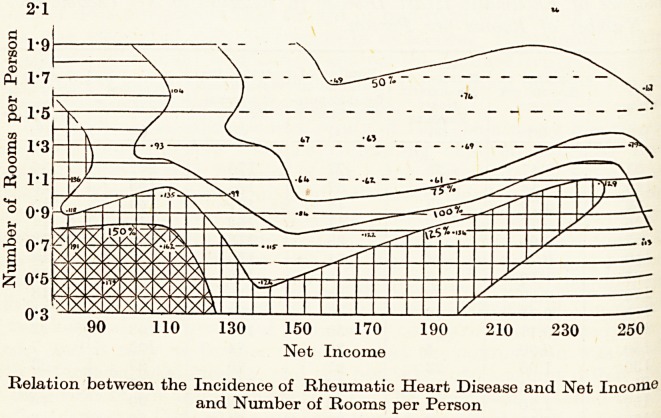# Social and Economic Conditions and the Incidence of Rheumatic Heart Disease
*The present study was suggested by Professor Bruce Perry of the Department of Medicine, Bristol University, who also found which Bristol families contained children suffering from rheumatic heart disease. The data upon which the study is based were collected by the Bristol Social Survey under the guidance of Mr. Herbert Tout. The writer is responsible for analysing the data and preparing the results for publication. Grateful acknowledgment is due to the Medical Research Council and the Colston Research Society for financial assistance, and to the University of Bristol for the facilities afforded for the investigation. Professor M. Greenwood and Dr. J. O. Irwin read the paper in draft and made valuable suggestions.


**Published:** 1943

**Authors:** G. H. Daniel


### SOCIAL AND ECONOMIC CONDITIONS AND THE INCIDENCE OF RHEUMATIC HEART DISEASE.*

BY

G. H. DANIEL, D.Phil.

[Reprinted with some omissions from the *Journal of the Royal Statistical Society*, Vol. CV., Part III., 1942, by kind permission of the Society.]

### INTRODUCTION.

DIFFERENCES in health between sections of a population may be reasonably considered as due to differences between them in their hereditary make-up, geographical distribution, or economic and social conditions of life. The latter are likely to affect health in several ways, since they include the nature and amount of nutrition which members of the population obtain, the adequacy of their housing accommodation and clothing, the amount of exposure and toil to which they are subjected and the degree of medical attention which they receive.

The difficulties in the way of isolating and measuring the factors which determine the incidence of disease are considerable. The phenomena in question are not under the control of the investigator, and the only information which can be secured for analysis is that relating to the existing differences between sections of the population in their constitution, distribution, conditions of life and the incidence of the disease. There is in the first place, therefore, the difficulty of ascertaining which of the many other points of difference are casually associated with the differences in incidence and are specific to the disease. It would be of little use to relate incidence to such an omnibus condition as the degree of urbanization, while, on the other hand, a relation established between the disease and a certain nutritional deficiency would be of practical value. In the second place, a causal relation existing between the incidence of the disease and any one factor—for example, nutrition—is likely to be obscured by the effects of other agencies, such as clothing or housing. It is necessary, therefore, to go beyond the *prima facie* relation between incidence and a particular factor, and to study, as far as possible, the relationship between them when other determining factors are held constant.

* The present study was suggested by Professor Bruce Perry of the Department of Medicine, Bristol University, who also found which Bristol families contained children suffering from rheumatic heart disease. The data upon which the study is based were collected by the Bristol Social Survey under the guidance of Mr. Herbert Tout. The writer is responsible for analysing the data and preparing the results for publication. Grateful acknowledgment is due to the Medical Research Council and the Colston Research Society for financial assistance, and to the University of Bristol for the facilities afforded for the investigation. Professor M. Greenwood and Dr. J. O. Irwin read the paper in draft and made valuable suggestions.

It appears from the literature on the aetiology of rheumatic disease that, so far, these difficulties have not been very successfully overcome, and although there is a substantial amount of published work on the subject, the conclusions which can be safely drawn are limited. Several writers have pointed to the existence of large differences in the incidence of rheumatic disease between children in private and children in State schools, between patients attended in private practice and hospital patients, and between children in poor-law schools and those in state schools.* The two populations in the last of these comparisons were drawn from a similar social and economic class, and may have had a similar hereditary background. But in the other two comparisons the discrepancy in incidence could clearly have been due to differences in hereditary constitution, geographical distribution, or social and economic conditions. The comparisons merely show that these factors between them gave rise to marked differences in incidence and that, on the whole, the poorer classes were associated with the higher incidence.

Other writers have attempted to relate the incidence of rheumatic disease to particular agencies, such as poverty (see, for example, Faulkner and White, 1924, Maddox, 1937, and Miller, 1928), nutrition (Miller and Wilson, 1939, and Vining, 1928), overcrowding (Perry and Roberts, 1937), damp (Coates and Thomas, 1925, Coombs, 1927, Miller, 1926, Newsholme, 1895, Thomson, 1926, and Young, 1925), the distribution of vermin (Clarke, 1928), industrialization (Young, 1921), urbanization (Atwater, 1927, and McCulloch, 1928), and heredity (Hill, 1928, Hill and Allan, 1929, and Sutton, 1928). The Medical Research Council Report of 1927 dealt with the effects of maternal care, subjection to exposure, parents' character, fathers' occupation, income, housing, sanitation, and elevation.

But many of these factors have been inadequately considered in relation to their likely effect on the disease, and others have been so broadly defined as to be useless for practical purposes. There is no obvious reason why parents' character should affect the susceptibility of children to disease, except in so far as it has some influence on the provision of the necessities of life in their homes, and thus, indirectly, affects the health of the children. Nor is it of value to find a relation between incidence and the degree of industrialization. Such a relation expresses only the sum effects of a great many specific factors associated together under industrial conditions, some affecting the disease in one way and some in other ways.

In no case does an effort appear to have been made to probe below the surface relations between the incidence of rheumatic disease and the conditions believed to affect it. The net relations—that is, the effects when other factors are constant—have not been examined. Thus Maddox (1937) plotted the location of known cases of rheumatic disease on maps showing climate, relief, distribution of underground water, and poverty, in an attempt to relate the disease to each of these conditions. But, even if the distribution of cases had corresponded closely with that, say, of water, this could have been because the total population, rheumatic and non-rheumatic, was also so distributed,

* See Faulkner and White (1924), Miller (1928), and Medical Research Council (1927). A review of the whole literature is given by Paul (1930).

because the people living near the water-courses were the poor, malnourished and susceptible classes, or because infective agencies were concentrated there.

Moreover, the methods employed in establishing relationships have frequently been not only limited but unreliable. Thus Miller concluded that dampness was a determining factor merely from an estimate of the number living in damp houses among 196 persons suffering from the disease. The Medical Research Council Report of 1927 sought to evaluate the effects of social and economic conditions by comparing the living conditions of 721 families containing children under hospital treatment for rheumatism with those of 200 families containing children attending hospital on account of non-rheumatic disease. These 200 families, however, included among their other members 43 cases of rheumatism. And, like the rheumatic families, they were families of the hospital class, and belonged presumably to the poorer sections of the community. It is quite possible that their diseases as well as that of the rheumatic families were related to adverse social and economic circumstances. In that case comparison of their conditions as made in the Report of the Medical Research Council could not be expected to reveal the importance of those conditions. The rheumatic families should be compared, not with families suffering from other diseases, but with the total population.

There can be little doubt that an important reason for the slow progress made in the study of the aetiology of rheumatic disease has been the cost of obtaining quantitative data about the condition of a reasonably large number of rheumatic and non-rheumatic families. To a limited extent such information was collected by the University of Bristol Social Survey in 1937. Although it was not the primary object of this Survey, advantage was taken of it to enquire into the relation between the social and economic conditions of life of the Bristol population and the incidence of the disease.

*The Bristol Data.*

In the summer of 1937 the University of Bristol, in conjunction with the Colston Research Society, investigated the circumstances of a random sample of the Bristol working-class population.* The families considered were those of manual workers, and black-coated workers with incomes not exceeding £5 per week, and information was secured from one family in twenty-two. It was clear that some of the aspects of social and economic conditions about which information was obtained were likely to affect susceptibility to disease. The opportunity was, accordingly, taken of collecting the same information about Bristol families with children suffering from rheumatic disease.

Rheumatic heart disease was chosen as the criterion of rheumatic infection, in order to reduce to the minimum doubts as to correct diagnosis. All cases of working-class children of school age—that is, between five and fourteen years of age—who had shown signs of active rheumatic infection during the eighteen months preceding or covering the period of the Social Survey were investigated for the study. They included the cases discovered by the School Medical Officers during their

* See Tout (1938).

routine examination and all cases receiving treatment at the two large voluntary hospitals, the children's hospital and the municipal hospitals. The only children not included were those receiving attention from private practitioners, but these were almost certainly above the income limits of the section of the population covered by the enquiry. A small number of the families containing rheumatic children were later found to be middle-class, and a few had moved from Bristol or refused information. The total number of families containing children five to fourteen years of age suffering from rheumatic heart disease and from whom information was obtained was 341.

From the sample of the entire working-class population, all families with children five to fourteen years of age were abstracted for comparison with the rheumatic population. The total number of such families was 1,424.

The information collected for both the rheumatic families and the sample of the total population, and which appeared likely to be related to the incidence of rheumatic disease, dealt with income, housing accommodation, membership of a doctor's club, and the receipt of meals or milk at school.

The size of the family income may be expected to affect the children's susceptibility to disease in three ways: the amount of money spent on food may limit the adequacy of their nutrition; the amount spent on clothes and fuel may be related to the degree of exposure ; and the outlay on rent and rates may largely determine the nature of their housing accommodation. The Social Survey secured information about the total earnings of each member of the family for the week preceding the date of investigation, and note was made of such items of income as gifts, free clothing, unemployment benefit, and pensions. The outgo on rent and rates, travelling expenses, insurance, trade union subscriptions, and other regular payments was also ascertained. Other expenditures were not investigated, but subtracting the outgo on fixed items from the total family income leaves a residue which was mainly laid out on food, clothes and fuel.* Lacking more detailed information, the figures for this residue may be related to the incidence of the disease.

It is not, of course, the total family income which is likely to affect susceptibility to disease, as much as the income of the family relative to its needs. It is necessary to adjust the total income according to the size of the family and the age and sex of its members. The minimum needs of each family were evaluated by the Social Survey† according to the age and sex of each member of the family, the standards adopted following, with small modifications, those laid down by George (1937). The net income for each family was expressed as a percentage of its minimum needs, and it is this percentage which is related in the present paper to the incidence of rheumatic heart disease.

* According to the Ministry of Labour's enquiry in 1937 into the expenditure of working-class households, the income left after paying for rent, rates, travelling, insurance and subscriptions was spent as follows :—

| | |
|---|---|
| Food .. .. .. | 51 per cent. |
| Clothes .. .. .. | 14 ,, |
| Fuel and Light .. .. | 10 ,, |
| Other items .. .. | 25 ,, |

See the *Ministry of Labour Gazette*, December, 1940.

† See Tout (1938).

Expenditure on rent and rates is known, but its effect on children's susceptibility to disease is unlikely to be close, since rent and rates depend largely on conditions such as location and relations between tenant and landlord which may be little connected with the adequacy of the accommodation from the health point of view. Moreover, because many houses are owner-occupied, the amount paid in rent and rates is not available for all families. The Social Survey found for each family the number of living-rooms and bedrooms occupied by it, and whether or not it possessed a kitchen and bathroom. The total number of these rooms used by each family, divided by the number of persons using them, was taken therefore as a more convenient as well as a more accurate index of housing accommodation.

It was also recorded on each of the Survey cards whether any of the living-rooms or bedrooms were situated in a basement, whether the families were members of a doctor's club, and whether the children received meals or milk at school. It was considered that each of these attributes might be related to susceptibility; the use of a basement room because of dampness or lack of light and ventilation, membership of a doctor's club because of the greater opportunities afforded by it for medical attention, and receipt of school meals or milk because of its supplementation of family income. Each of the three attributes enables the population to be divided into two classes, according to possession or non-possession of it, and all three attributes together enable the families to be divided, as shown in Table III, into eight classes.

The incidence of the disease was measured and related to these social and economic conditions by taking each class into which the population could be divided on the basis of net income, housing accommodation, etc., and finding the number of families falling into it among the rheumatic families on the one hand and among the sample of the total population on the other.

The number of rheumatic families in each class was divided by the number of families from the sample of the total working-class population in the same class and, to facilitate comparison of incidence between the classes, the result was expressed as a percentage of the proportion between all 341 rheumatic families and 1,424 families in the total population sample. This gave for each class the incidence of the disease as a percentage of the average incidence among the entire population studied.

The Bristol data thus yields the following information:—

(*a*) Incidence of rheumatic heart disease in each group of working-class families as a percentage of the average incidence for all Bristol working-class families. This will be taken as the dependent variable ($Y$) and for the sake of brevity it will be referred to as the "incidence."

(*b*) Income available for expenditure on food, clothes and fuel as a percentage of minimum needs. This independent variable ($X_1$) will be referred to simply as the "net income."

(*c*) Number of rooms per person ($X_2$).

(*d*) Use of a basement room, receipt of meals or milk at school, and membership of a doctor's club.

*Total Relations between Economic and Social Conditions and the Incidence of Rheumatic Heart Disease.*

The relation between rheumatic heart disease and conditions of life may be studied by examining, first of all, the association between incidence and each one in turn of the independent variables which have just been described.

In Table I the families are divided among eight net income groups. The second column shows the number of rheumatic families in each group, and the third column the total number of working-class families. The figures of incidence set out in the following column were calculated, as already explained, by dividing the figures in the second column by those in the third and expressing the results as percentages of the average proportion for the whole population.

The figures show a marked relation between net income and incidence; families with incomes below their minimum needs included

TABLE I

*Incidence of Rheumatic Heart Disease in Relation to Net Income.*

| Net Income (as a % of minimum needs). (1) | Families with Rheumatic Heart Disease. (2) | Families in Sample of Total Working-Class Population. (3) | Incidence of Disease (as a % of average incidence). (4) | Expected Incidence.* (5) |
|---|---|---|---|---|
| Under 100.. .. .. | 77 | 232 | 139 | 142 |
| 100–120 .. .. .. | 54 | 152 | 148 | 122 |
| 120–140 .. .. .. | 51 | 191 | 112 | 108 |
| 140–160 .. .. .. | 33 | 192 | 72 | 97 |
| 160–180 .. .. .. | 37 | 192 | 81 | 88 |
| 180–200 .. .. .. | 31 | 149 | 87 | 81 |
| 200–220 .. .. .. | 17 | 93 | 76 | 75 |
| 220 and over .. .. | 41 | 223 | 77 | 70 |
| Total .. .. | 341 | 1,424 | — | — |

* Estimated from $Y = 4{,}291\ X_1^{-0 \cdot 757}$.

approximately 40 per cent. more cases of rheumatic heart disease than did the average working-class families, and approximately twice as many as the working-class families in the highest net income group. These differences in incidence could be due, at least partly, to the smallness of the samples, the proportion of rheumatic heart families in each group being known only from the limited experience of eighteen months, and the distribution of all the working-class families being based on a random sample of one family in twenty-two. The author calculates that there is a probability of less than one in a hundred that the differences can be explained on this ground.

The relation between incidence and the number of rooms per person may be examined in the same way. Table II presents a classification of the data with respect to rooms per person like that given in Table I with respect to net income. The differences in incidence shown in the fourth column are highly significant. These differences show a clearly defined inverse relation between incidence and rooms per person.

Families with less than 0.6 of a room per person are shown by this table to have had almost four times as many cases as those with 1.8 rooms or more.

TABLE II.

*Incidence of Rheumatic Heart Disease in Relation to the Number of Rooms per Person.*

| Rooms per Person. (1) | Families with Rheumatic Heart Disease. (2) | Families in Sample of Total Working-Class Population. (3) | Incidence of Disease (as a % of average incidence). (4) | Expected Incidence.* (5) |
|---|---|---|---|---|
| Under 0.6 .. .. | 52 | 130 | 167 | 187 |
| 0.6–0.8 .. .. .. | 69 | 193 | 149 | 136 |
| 0.8–1.0 .. .. .. | 53 | 194 | 114 | 107 |
| 1.0–1.2 .. .. .. | 73 | 335 | 91 | 89 |
| 1.2–1.4 .. .. .. | 59 | 303 | 81 | 76 |
| 1.4–1.6 .. .. .. | 10 | 71 | 59 | 66 |
| 1.6–1.8 .. .. .. | 19 | 138 | 58 | 59 |
| 1.8 and over .. .. | 6 | 60 | 42 | 53 |
| Total .. .. | 341 | 1,424 | — | — |

* Estimated from $Y = 95.1 \,.\, X_2^{-1.037}$.

Table III displays the eight classes into which the population may be divided according to receipt or non-receipt of meals or milk at school,

TABLE III.

*Incidence of Rheumatic Heart Disease in Relation to Membership of a Doctor's Club, Receipt of School Meals or Milk, and the Use of Basement Rooms.*

| Classification of Families. | Members of a Doctor's Club. | | | | Not Members of a Doctor's Club. | | | |
|---|---|---|---|---|---|---|---|---|
| | Receiving School Meals or Milk. | | Not Receiving School Meals or Milk. | | Receiving School Meals or Milk. | | Not Receiving School Meals or Milk. | |
| | No Basement Rooms. (1) | Basement Rooms. (2) | No Basement Rooms. (3) | Basement Rooms. (4) | No Basement Rooms. (5) | Basement Rooms. (6) | No Basement Rooms. (7) | Basement Rooms. (8) |
| Families with Rheumatic Heart Disease .. .. | 4 | — | — | — | 224 | 23 | 84 | 6 |
| Families in Sample of Total Population .. .. | 51 | 2 | 5 | — | 980 | 60 | 307 | 19 |
| Incidence .. .. .. | 33 ± 21 | —* | —* | —* | 95 ± 5 | 160 ± 51 | 114 ± 16 | 132 ± 83 |

* Insufficient data.

membership or non-membership of a doctor's club, and use or non-use of a basement for living or sleeping purposes. The advantage of this manifold classification is that it shows the relation between incidence and each attribute independently of the other attributes. Unfortunately, the data are too scanty to enable full use to be made of the table, there being hardly any observations in the second, third and fourth columns. In some of the remaining columns the numbers of families are so small that comparison must necessarily be tentative and no certain conclusions can be drawn.

Because it is necessary to consider the differences in incidence between each column, the figures of incidence have had attached to them their standard errors. These errors arise from the fact that both the number of families in the total working population and the number of rheumatic heart families in each column are known only from two limited samples drawn at random.

Comparison of columns (1) and (5) suggests that membership of a doctor's club was associated with a low incidence. Taking columns (5) and (7) for comparison on the one hand, and columns (6) and (8) on the other, it does not appear that receipt or non-receipt of school meals or milk was associated with differences in incidence. Examining column (5) against column (6), and also (7) against (8), families living or sleeping in basements had a higher incidence than those which did not, but the differences are not statistically significant.

The Medical Research Council study of 1927, as already mentioned, compared hospital-class families containing children suffering from rheumatic disease with families of the same class suffering from other diseases. The two groups of families were divided into three classes according to their average total income. No direct relation between the disease and income was discovered, and incidence was in fact found to be lowest among the families with less than 35s. per week and highest among those in the middle income group with 35s. to 59s., although the differences were in each case not significant. Criticism of the choice of two hospital-class groups of families for comparison has already been made, and it may be further objected that the comparison was made with respect to total income, and did not take into account differences in the needs of the families concerned. The conclusion reached in the Medical Research Council Report that there is no direct relation between the occurrence of rheumatic heart disease and poverty, and even the finding that incidence is highest in the middle income ranges of the working-class population, has been widely accepted in subsequent literature. The Bristol data show, however, that after adjustment has been made for needs there is a simple inverse relation between incidence and the income left after paying for rent, rates, travelling and other fixed expenses.

The Medical Research Council Report also dealt with the number of rooms used by the families examined and with the use of basement rooms. No significant relation was found between either of these and the disease. But the present data suggest that there is a close and important relation between incidence and the number of rooms per person, and there may also be a relation between it and the employment of basement accommodation.

The present findings agree with those of Perry and Roberts (1937). These writers related the number of cases of rheumatic heart disease per 100,000 population with the density of persons per room in twenty-three Bristol City Wards during the three years 1927–30. They found a simple and significant relation and obtained a coefficient of correlation of 0.84.

*Partial Relations between Social and Economic Conditions and the Incidence of Rheumatic Heart Disease.*

The question arises whether the relations found between incidence and each in turn of the three independent variables exist when the remaining two independent variables are held constant.

In Table IV are shown twenty-seven figures of incidence and the corresponding values of net income and rooms per person. To prepare it, the rheumatic heart families and the families in the total working-class population were divided among the 120 classes obtained by taking

TABLE IV.

*Incidence of Rheumatic Heart Disease in Relation to Net Income and Number of Rooms per Person.*

| Net Income (1) | Number of Rooms per Person (2) | Actual Number of Rheumatic Heart Families (3) | Number of Families in Total Population Sample (4) | Actual Incidence (as % of average incidence) (5) | Expected Incidence * (6) | Expected Number of Rheumatic Heart Families * (7) |
|---|---|---|---|---|---|---|
| 93 | 0.47 | 33 | 77 | 179 | 202 | 37 |
| 140 | 0.48 | 11 | 37 | 124 | 168 | 15 |
| 82 | 0.70 | 22 | 48 | 191 | 159 | 18 |
| 110 | 0.70 | 12 | 31 | 162 | 141 | 11 |
| 82 | 0.90 | 11 | 39 | 118 | 132 | 12 |
| 140 | 0.70 | 14 | 51 | 115 | 127 | 16 |
| 83 | 1.10 | 15 | 46 | 136 | 113 | 13 |
| 110 | 0.99 | 22 | 68 | 135 | 109 | 18 |
| 170 | 0.78 | 16 | 55 | 122 | 109 | 14 |
| 196 | 0.77 | 16 | 50 | 134 | 103 | 12 |
| 150 | 0.90 | 6 | 30 | 84 | 103 | 7 |
| 130 | 1.05 | 23 | 97 | 99 | 97 | 23 |
| 254 | 0.74 | 10 | 37 | 113 | 96 | 8 |
| 107 | 1.30 | 19 | 85 | 93 | 90 | 18 |
| 150 | 1.10 | 6 | 39 | 64 | 89 | 8 |
| 170 | 1.10 | 7 | 47 | 62 | 84 | 9 |
| 190 | 1.10 | 7 | 48 | 61 | 81 | 9 |
| 150 | 1.33 | 9 | 56 | 67 | 77 | 10 |
| 111 | 1.67 | 10 | 40 | 104 | 74 | 7 |
| 239 | 1.10 | 16 | 52 | 129 | 73 | 9 |
| 170 | 1.35 | 10 | 66 | 63 | 72 | 11 |
| 200 | 1.30 | 11 | 67 | 69 | 70 | 11 |
| 249 | 1.30 | 10 | 53 | 79 | 64 | 8 |
| 162 | 1.77 | 4 | 34 | 49 | 62 | 5 |
| 199 | 1.62 | 9 | 51 | 74 | 59 | 7 |
| 255 | 1.65 | 7 | 62 | 47 | 53 | 8 |
| 224 | 2.11 | 5 | 58 | 36 | 47 | 6 |

* Estimated from $Y = 757.3\ X_1^{-0.414}.\ X_2^{-0.742}$.

ten classes of net income and twelve of rooms per person. Classes containing not more than thirty families from the sample of the total population were combined with adjacent classes until they had the requisite number of families. For groups made up in this way, the values of net income and rooms per person were found by taking the average of the class-marks of the constituent sub-groups, each class-mark being weighted by the number of families belonging to the sample of the total population. The incidence in each of the twenty-seven classes was measured, as already described, by dividing the number of rheumatic families in each class by the total number of families in it, and expressing the result as a percentage of the proportion between all the 341 rheumatic families and the 1,424 families in the sample of the working-class population.

The nature of the relation between incidence and both net income and number of rooms per person is shown graphically below. The twenty-seven points marked in the figure represent the values in the table, and each point has been located with reference to the values for net income and rooms per person. Contour lines have been drawn in

Relation between the Incidence of Rheumatic Heart Disease and Net Income and Number of Rooms per Person relation to the values of incidence for each point. These lines show that the incidence of the disease was highest when net income was low and when, at the same time, there were few rooms per person. At any given income level, the incidence tended to vary inversely with the number of rooms per person; conversely, with a given number of rooms per person, the incidence tended to increase as net income diminished.

After calculating the relations between these variables, the author finds that, when there was no difference in the number of rooms per person, a given percentage difference in net income was accompanied by a difference of 0·414 ± 0·144 times as much in incidence. On the other hand, among families with the same net income, those with a given percentage of rooms per person more than the others had 0·742 ± 0·136 times less incidence.

The association found in Table III between incidence and membership of a doctor's club, receipt of meals or milk at school, and use of a basement room could perhaps be due to the existence of relationships between these attributes and net income or rooms per person, and between the latter and incidence. Of the eight classes into which the families can be divided according to possession or non-possession of the three attributes, the five which contain a reasonable number of families are shown again in Table V. The top row gives the average net income of the families in each class ; the second shows the average number of rooms per person ; and in the third row the figures of incidence obtained in Table III are repeated.

TABLE V.

*Incidence of Rheumatic Heart Disease in Relation to Membership of a Doctor's Club, Receipt of School Meals or Milk, and Use of Basement Rooms (after Approximate Removal of the Effects of $X_1$ and $X_2$).*

| Classification of Families. | Members of a Doctor's Club. | Not Members of a Doctor's Club. | | | |
|---|---|---|---|---|---|
| | Receiving School Meals or Milk. | Receiving School Meals or Milk. | | Not Receiving School Meals or Milk. | |
| | No Basement Rooms. (1) | No Basement Rooms. (5) | Basement Rooms. (6) | No Basement Rooms. (7) | Basement Rooms. (8) |
| Net Income .. .. .. | 161 | 160 | 146 | 160 | 161 |
| Rooms per Person .. .. | 1.12 | 1.12 | 0.94 | 1.16 | 1.14 |
| Actual Incidence .. .. | 33 | 95 | 160 | 114 | 132 |
| Incidence Expected from Values of Net Income and Rooms per person .. | 100 | 100 | 118 | 97 | 98 |
| Incidence Adjusted for Differences in Net Income and Rooms per Person.. .. | 33 | 95 | 136 | 118 | 135 |

Incidence in class (6) was shown in Table III to have been unexpectedly high. The present table indicates that the families in this class had on the whole a relatively low net income and comparatively few rooms per person, and there are also some differences with respect to these conditions between the other classes. But when incidence is adjusted for these differences, it is seen that the relation depicted in Table III is not substantially altered. Looking at columns (1) and (5), families which did not belong to a doctor's club appear to have had a substantially higher incidence than those which did. Comparing columns (5) and (6) and also (7) and (8), the use of a basement room is seen to have been associated with a high incidence. On the other hand, columns (5) and (7), and columns (6) and (8), do not show a clear association between the disease and the receipt of school meals or milk.

Finally, a recalculation made by the author of the relationship between $Y, X_1$ and $X_2$, showed no indication that the one originally found reflected the association between incidence and the three attributes.

### CONCLUSION.

Review of the existing literature on the effect of social and economic conditions on the incidence of rheumatic heart disease shows that most of the analyses made have been limited in scope and productive of few conclusions. It has, however, been demonstrated that marked differences in incidence exist between various social classes, and it has also been shown (Perry and Roberts, 1937) that incidence bears a simple and direct relation to the degree of overcrowding.

The information gathered about the living conditions of the Bristol working-class population shows that :—

(*a*) Differences in incidence of rheumatic heart disease between sections of this population were associated with differences in the family income available after paying for rent, rates, travelling and other fixed items expressed as a percentage of minimum needs.

(*b*) Considerable differences in incidence corresponded closely with the variation in the number of rooms used by each family divided by the number of persons in the family.

(*c*) Families which belonged to doctors' clubs suffered less from the disease than did the remainder of the population.

(*d*) There is an indication, though the results are not statistically significant, that families which lived or slept in basement rooms included more cases of the disease than other families.

(*e*) Each one of these relations was true independently of the others.

To what extent these findings, based on the Bristol 1937 experience, apply to the population of the whole country is unknown. But Bristol is a large town, and there is no apparent reason why the relations found should be confined to it.

If these relationships are causal, a considerable reduction in the incidence of rheumatic heart disease could be effected by improvements in living conditions. The figures in Table V enable an estimate to be made of the effect of raising the net income and increasing the housing accommodation of the poorer families. The values in column (7) sum up the combined influence of net income and rooms per person, and may be taken as a measure of the poverty of the families in both respects. A calculation was made by arranging the twenty-seven sets of figures in the table in order according to the expected incidence. The number of families given in columns (3) and (4) were then cumulated, so that the total number of families and the number of rheumatic heart families with living standards equal to, or above, a given level, as measured in column (7), were known. It was then possible to find the number of cases of the disease to be expected if the entire population had had the same living conditions as the families considered, to calculate the percentage reduction in incidence, and, by graphical interpolation, to find the figures below :—

| Percentage of the Population with the lowest Net Income and fewest Rooms per Person. | Percentage Reduction in Incidence of Disease due to Improvement in Standards of the Poorest Families. |
|---|---|
| 10 | 8 |
| 30 | 21 |
| 50 | 29 |
| 70 | 37 |
| 90 | 49 |

Thus, if the standards of the 30 per cent. of the Bristol working-class population with the most inadequate incomes and housing accommodation were raised to the average level of the rest of the working-class population, a decrease of 21 per cent. in the number of cases of rheumatic heart disease could be expected. And if standards were raised to the level of the highest 10 per cent. of all working-class families, the incidence of the disease would be roughly halved.

Whether income and housing conditions actually determine the incidence of the disease in this way is not, however, established by the statistical relationships which have been revealed in preceding sections of this paper. It is necessary to consider whether these relations can be accounted for in other ways.

The data collected by the Bristol Social Survey throw no light on the effects of differences in geographical environment or in hereditary constitution. But the variations in altitude, soil, climate, etc., within the City of Bristol are minute, and could hardly account for the relationships we have found. Explanation in terms of heredity is equally difficult, since it would require that families with an inherited tendency towards rheumatism should tend to have low net incomes, and also to have poor housing conditions and refrain from joining doctors' clubs.

The present information relates only to families with children of school age. All of these children attended State schools and, outside their homes, their environment could have differed comparatively little. Thus, not only are the present results unlikely to have been due to the influence of hereditary or geographical agencies, but the possible effects of occupational differences, which in the case of adults might have complicated those of income and housing, have been avoided.

Again, this investigation has been concerned not with the individual children suffering from rheumatic heart disease, but with the families containing such children. Rheumatic disease may be infective. If so, the proportion of children in a family suffering from it would tend to be greater in large families than in small. Since size of family is an important cause of poverty and inadequate housing accommodation, there is, therefore, a possibility that a correlation disclosed between incidence and net income or between incidence and rooms per person could be due merely to the increased likelihood of infection in large families and the tendency for such families to have low standards of living. This possibility has been guarded against by confining examination to family units and giving those with three children suffering from the disease the same weight as those with only one.

The independent variables which have been considered are of broad scope, and exactly how they affect incidence it is not possible to say. Net income, for instance, may be related to the disease, because it limits the consumption of certain essential foodstuffs, or because it determines the adequacy of clothing or heating. New and detailed enquiries would be necessary to isolate the specific factors involved. What the present investigation shows is that rheumatic heart disease appears to be in large part a social disease, and that considerable differences in its incidence are attributable to conditions associated with: (*a*) the income available after deduction of rent, rates, travelling and other fixed payments; (*b*) the number of rooms available per person; (*c*) the membership of doctors' clubs; and, (*d*) though less certainly, the use of basement rooms.

### REFERENCES.

Atwater, R. M. (1927). Studies in the Epidemiology of Acute Rheumatic Fever and Related Diseases in the United States, based on Mortality Statistics. *Am. J. Hygiene*, VII, 343.

Clarke, J. T. (1928). The Pathogenesis of Rheumatic Fever in its Climatological Relationship to a Possible Insect Carrier. *Proc. Roy. Soc. Med.* Sect. Epidemiol. and State Med., XXI, 1004.

Coates, V., and Thomas, R. E. (1925). Rheumatic Infection in Childhood. *Lancet*, 1925, II, 326.

Coombs, C. F. (1927). Rheumatic Infection of Childhood. *Lancet*, 1927, I, 579 and 634.

Faulkner, J. M., and White, P. D. (1924). The Incidence of Rheumatic Fever, Chorea and Rheumatic Heart Disease with Especial Reference to its Occurrence in Families. *J. Am. Med. Ass.*, LXXXIII, 425.

George, R. F. (1937). A New Calculation of the Poverty Line. *Jour. Roy. Stat. Soc.*, 1937, C, 74.

Hill, N. G. (1928). The Rheumatic Child. *Brit. Jour. Child. Dis.*, XXV, 270.

Hill, N. G., and Allan, M. (1929). The Rheumatic Type. *Brit. Med. Jour.*, 1929, II, 499.

Maddox, K. (1937). Metropolitan and Rural Incidence and Distribution of Acute Rheumatism and Rheumatic Heart Disease in New South Wales. *Med. Jour. Australia*, 31st March, 394, 20th March, 425, 27th March, 464, 3rd April, 499.

McCulloch, H. (1928). Rheumatic Heart Disease : Its Importance as a Disease of Children. *Jour. Am. Med. Ass.*, XC, 2073.

Medical Research Council (1927). Child Life Investigations : Social Conditions and Acute Rheumatism. *Med. Res. Coun. Special Report Series* No. 114.

Miller, R. (1926). Report on Rheumatic Disease in Children : I. Report on the Environmental and Other Predisposing Causes of Rheumatic Infection. *Suppl. Brit. Med. Jour.*, 3rd July, 1926.

Miller, R. (1928). Some Public Health Aspects of Juvenile Rheumatism. *Proc. Roy. Soc. Med.* (Sect. Epidemiol. and State Med.), XXI, 997.

Miller, S., and Wilson, J. V. (1939). Nutrition and Diet in Rheumatism. *Med. Press and Circ.*, CCI, Nos. 16 and 17, 390 and 411.

Newsholme, A. (1895). The Natural History and Affinities of Rheumatic Fever: A Study in Epidemiology. The Milroy Lecture, *Lancet*, 1895, I, 589 and 657.

Paul, J. R. (1930). The Epidemiology of Rheumatic Fever. Printed by the *Metropolitan Life Insurance Company*.

Perry, C. B., and Fraser Roberts, J. A. (1937). A Study on the Variability in the Incidence of Rheumatic Heart Disease within the City of Bristol. *Suppl. Brit. Med. Jour.*, 1937, II, 154.

Sutton, L. P. (1928). Observations on Certain Aetiological Factors in Rheumatism. *Am. Heart Jour.*, IV, 145.

Thomson, A. P. (1926). A Study of Rheumatism in Children. *Birmingham Med. Rev.*, n.s. I, No. 7.

Tout, H. (1938). The Standard of Living in Bristol.

Vining, C. W. (1928). The Pre-Rheumatic Child. *Med. Jour. and Rec.*, CXXVIII, 351, 395 and 453.

Young, M. (1921). A Preliminary Study on the Epidemiology of Rheumatic Fever, *Jour. Hyg.*, XX, 248.

Young, M. (1925). The Geographical Distribution of Heart Disease in England and Wales and its Relation to that of Acute Rheumatism. *Lancet*, 1925, II, 590.

## Figures and Tables

**Figure f1:**